# Real-World Early Prostate-Specific Antigen Response Comparison Among Patients with Metastatic Castration Sensitive Prostate Cancer Treated with Apalutamide or Abiraterone Acetate in the United States

**DOI:** 10.36469/001c.170641

**Published:** 2026-10-30

**Authors:** Benjamin Lowentritt, Mehmet A. Bilen, Sabree Burbage, Charmi Patel, Frederic Kinkead, Carmine Rossi, Gordon Wong, Dominic Pilon, Neal Shore

**Keywords:** androgen receptor pathway inhibitors, castration-sensitive, comparative effectiveness, hormonal antineoplastic agents, hormone-sensitive, prostate-specific antigen, prostatic neoplasms

## Abstract

**Background:**

Despite the availability of several androgen receptor pathway inhibitors for the treatment of patients with metastatic castration-sensitive prostate cancer (mCSPC), there has been a lack of head-to-head randomized clinical trials to evaluate their comparative efficacy in this population.

**Objective:**

This real-world study provides a comprehensive and robust comparison of the decline in prostate-specific antigen ≥90% (PSA90) among patients with mCSPC who initiated apalutamide or abiraterone acetate in the United States.

**Methods:**

Clinical data were collected from US community urology practices by Precision Point Specialty Analytics and linked with administrative claims from the Komodo Health Solutions Research Database. Patients with mCSPC were included in apalutamide or abiraterone acetate cohorts based on their first dispensation/paid claim on or after September 17, 2019 (index date), and followed to the earliest of treatment discontinuation or switch, end of insurance/clinical activity, or end of data availability (Dec. 31, 2023). Inverse probability of treatment weighting balanced potentially confounding patient characteristics. PSA90 response by 6 months post index was compared between the cohorts using weighted Kaplan-Meier analysis and time to PSA90 response was compared using a weighted Cox model with a time-varying treatment effect.

**Results:**

The study included 1351 patients in the apalutamide cohort and 1003 patients in the abiraterone acetate cohort. By 6 months, 61.4% of the weighted apalutamide cohort and 45.9% of the weighted abiraterone acetate cohort achieved a PSA90 response. Compared with abiraterone acetate, patients treated with apalutamide were 73% more likely to achieve a PSA90 response at 6 months (weighted hazard ratio, 1.73; 95% confidence interval, 1.44-2.08; *P* < .001). The median time to PSA90 response was 3.8 and 8.7 months in the apalutamide and abiraterone acetate cohorts, respectively.

**Conclusions:**

The results of this study confirm that patients with mCSPC treated with apalutamide have significantly higher rates of PSA90 response compared with abiraterone acetate after 6 months of treatment. Real-world evidence highlights apalutamide as an important treatment option to improve the prognosis of patients with mCSPC.

## INTRODUCTION

With a continuously aging global population, the incidence of genitourinary tumors, such as prostate cancer (PC), is expected to rise over time.^1^ As the most frequently diagnosed cancer in men, more than 300 000 new cases of PC arise in the United States (US) each year.^2^ In approximately one-third of cases, PC is diagnosed in the nonlocalized setting and it is the second leading cause of cancer death with a 5-year survival as low as 38% for patients with metastasis.^3^ Although patients with metastatic PC may initially respond to treatment with androgen deprivation therapy (ADT), most eventually experience disease progression from metastatic castration-sensitive PC (mCSPC; also known as metastatic hormone-sensitive PC) to metastatic castration-resistant PC (mCRPC), which results in poorer prognosis.^4,5^ To delay disease progression and improve survival outcomes in patients with mCSPC, current clinical guidelines recommend ADT in combination with apalutamide, enzalutamide, or darolutamide, three androgen receptor pathway inhibitors (ARPIs) that block the competitive binding of androgen to the androgen receptor (AR) and inhibit AR nuclear translocation, DNA binding, and coactivator recruitment, or abiraterone acetate, an ARPI that blocks the conversion of steroid precursors to dehydroepiandrosterone to inhibit CYP17A1 in adrenal gland and tumor tissue,^6^ based on evidence from clinical trials showing superior efficacy of ARPIs relative to ADT alone.^7–11^

Despite the availability of several ARPIs for the treatment of patients with mCSPC, there has been a lack of head-to-head randomized clinical trials (RCT) to evaluate their comparative efficacy in this population. One network meta-analysis of RCT data suggested that apalutamide (which reduced the risk of death by 35% relative to ADT alone in the TITAN RCT^12^) and abiraterone acetate (which reduced the risk of death by 34% relative to ADT alone in the LATITUDE RCT^13^) may provide the largest benefit for patients with mCSPC relative to the other available ARPI treatments.^14^ Moreover, recent real-world evidence suggests that treatment with apalutamide may be associated with better long-term outcomes than abiraterone acetate for patients with mCSPC.^15^ Specifically, a real-world head-to-head analysis of nearly 4000 ARPI-naïve patients with mCSPC showed that apalutamide was associated with a 26% reduction in the risk of mortality by 24 months post-treatment initiation compared with abiraterone acetate.^15^

A growing body of evidence has also shown that among patients with PC, prostate-specific antigen (PSA) response, particularly the attainment of early (ie, by 6 months from treatment initiation) and deep PSA90 response (ie, ≥90% decrease in PSA level from the most recent pre-treatment PSA level), can be an important prognostic factor.^16–23^ Indeed, a single-center real-world study of approximately 100 patients with mCSPC showed that those treated with apalutamide experienced a deeper and more sustained reduction in PSA levels compared with abiraterone acetate, which resulted in a significant delay in disease progression.^24^ Also, a recent real-world study of patients treated with ARPIs found that patients who achieved an early PSA90 response (ie, by 6 months) had significantly lower rates of mortality in comparison to those who did not achieve an early response.^23^

However, a larger corpus of data is needed to provide the evidence necessary to determine the comparative benefit of these two treatments and guide decision-making in clinical practice. To that end, this study builds upon prior work evaluating the impact of apalutamide vs abiraterone acetate on PSA90 response among patients with mCSPC.^25,26^ In the prior analysis of real-world data by Brown et al, which was based on linked administrative insurance claims with electronic medical records (EMRs) from a consortium of community-based urology practices, apalutamide use was associated with a 68% greater likelihood of achieving PSA90 response by 6 months after treatment initiation compared with abiraterone acetate among patients with mCSPC.^26^ The goal of this retrospective study was to extend this analysis by using more robust contemporary linked clinical and claims data, with a sample that included more than 1000 patients in each treatment arm, to confirm the real-world effectiveness of apalutamide over abiraterone acetate in inducing an early and deep PSA response among patients with mCSPC.

## METHODS

### Data Sources

The retrospective study used EMR data obtained from Precision Point Specialty Analytics (PPS) that were collected in routine clinical care from >90 private, US community-based urology practices. To supplement this information with demographic and clinical variables, and to capture medications administered outside of urology care settings, insurance claims from the Komodo Health Solutions Research Database (KRD) were linked to the EMR data obtained from PPS. Data were obtained from both sources between September 17, 2018 (ie, 12 months preceding the approval date for apalutamide), and December 31, 2023.

PPS provides data on many valid PSA measurements as well as other laboratory and vital data that are not available through traditional administrative claims data sources. The data include information on patient demographics, diagnoses and dates of diagnoses, medication dispensations for next-generation ARPIs (ie, fill dates, amount dispensed, and dosage), medication and procedure information used to identify ADT, first-generation ARPIs (ie, bicalutamide, nilutamide, or flutamide), and other PC-related medications (ie, chemotherapies, poly ADP-ribose polymerase [PARP] inhibitors, immunotherapies, estrogens, radiopharmaceuticals, and bone antiresorptive therapy), and procedure information used to identify imaging tests (ie, bone scan, computerized tomography scans, and next-generation imaging). The PPS database includes robust data from urology practices that treat a large number of patients with mCSPC and is therefore representative of the patient distribution in the US.

The KRD data are sourced from a variety of payers and healthcare organizations and reflect over 320 million US patients across Medicaid, Commercial, and Medicare insurers. Data from both the open and closed claims KRD portions were used in this study as they include information on prescription fills, diagnoses, and procedures received in inpatient and outpatient settings, and billing and reimbursement information.

All data were de-identified and compliant with the requirements of the Health Insurance Portability and Accountability Act (HIPAA).

### Study Design and Sample Selection

A retrospective, longitudinal, cohort design was used for this comparative analysis of patients with mCSPC who initiated apalutamide or abiraterone acetate (**Figure 1**). The index ARPI treatment, which determined the mutually exclusive study cohorts, was defined by the earliest dispensation or paid pharmacy claim for apalutamide or abiraterone acetate on or after September 17, 2019 (ie, the approval date for apalutamide for mCSPC). The 12 months prior to the index date were defined as the baseline period, and the follow-up period spanned from the index date to the earliest of index treatment discontinuation (using a 90-day treatment gap to define treatment discontinuation), initiation of a radiopharmaceutical agent or a non-index ARPI (ie, apalutamide [if the index treatment was abiraterone acetate], abiraterone acetate [if the index treatment was apalutamide], enzalutamide, or darolutamide), end of claims activity or clinical activity (including death), or end of data availability (ie, Dec. 31, 2023).

**Figure 1.**
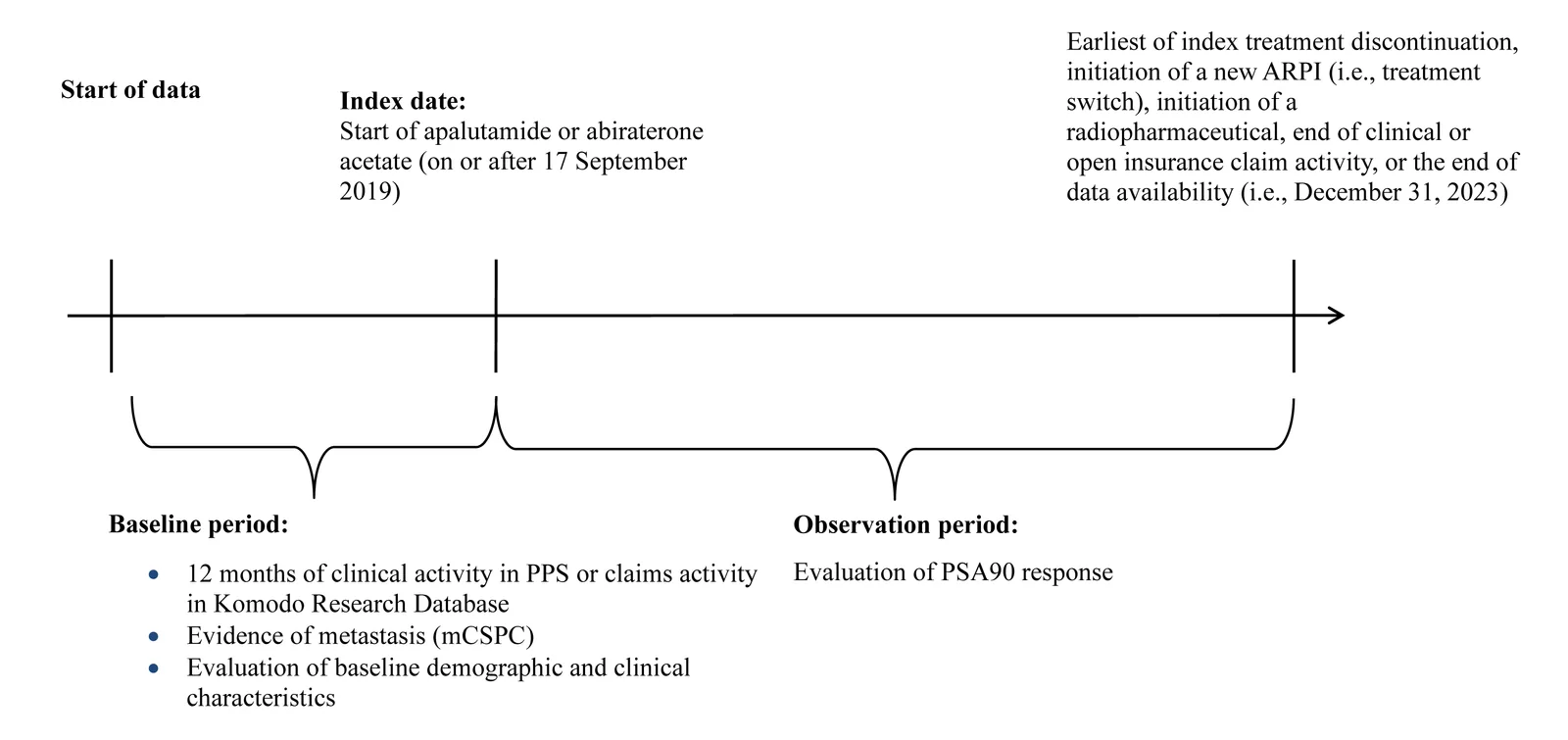
Study Design Abbreviations: ARPI, androgen receptor pathway inhibitor; mCSPC, metastatic castration-sensitive prostate cancer; PPS, Precision Point Specialty Analytics; PSA, prostate-specific antigen.

The study included adult patients with a diagnosis of mCSPC prior to or on the index date, which was identified through derived variables from PPS or diagnosis codes for metastasis in PPS and KRD. Patients were required to have a measurable follow-up period after the index date, ≥12 months of clinical activity in PPS or claims activity in KRD prior to the index date, and ≥1 PSA measurement in the 13-week period prior to or on the index date. Clinical activity was defined as the earliest observed record to last record in PPS, and claims activity was defined as the earliest observed claim to last claim in KRD. Patients with evidence of castration resistance, a diagnosis for other primary cancers during the 12-month baseline period, non-index ARPI, estrogen, chemotherapy (ie, etoposide, cabazitaxel, or carboplatin after docetaxel), immunotherapy, PARP inhibitor, or radiopharmaceutical use any time prior to or on the index date, or apalutamide or abiraterone acetate use prior to September 17, 2019, were excluded from the study. Patients were not required to use concurrent ADT to be included in the apalutamide or abiraterone acetate cohorts, and they were not required to use concurrent prednisone to be included in the abiraterone acetate cohort (**Figure 2**).

**Figure 2.**
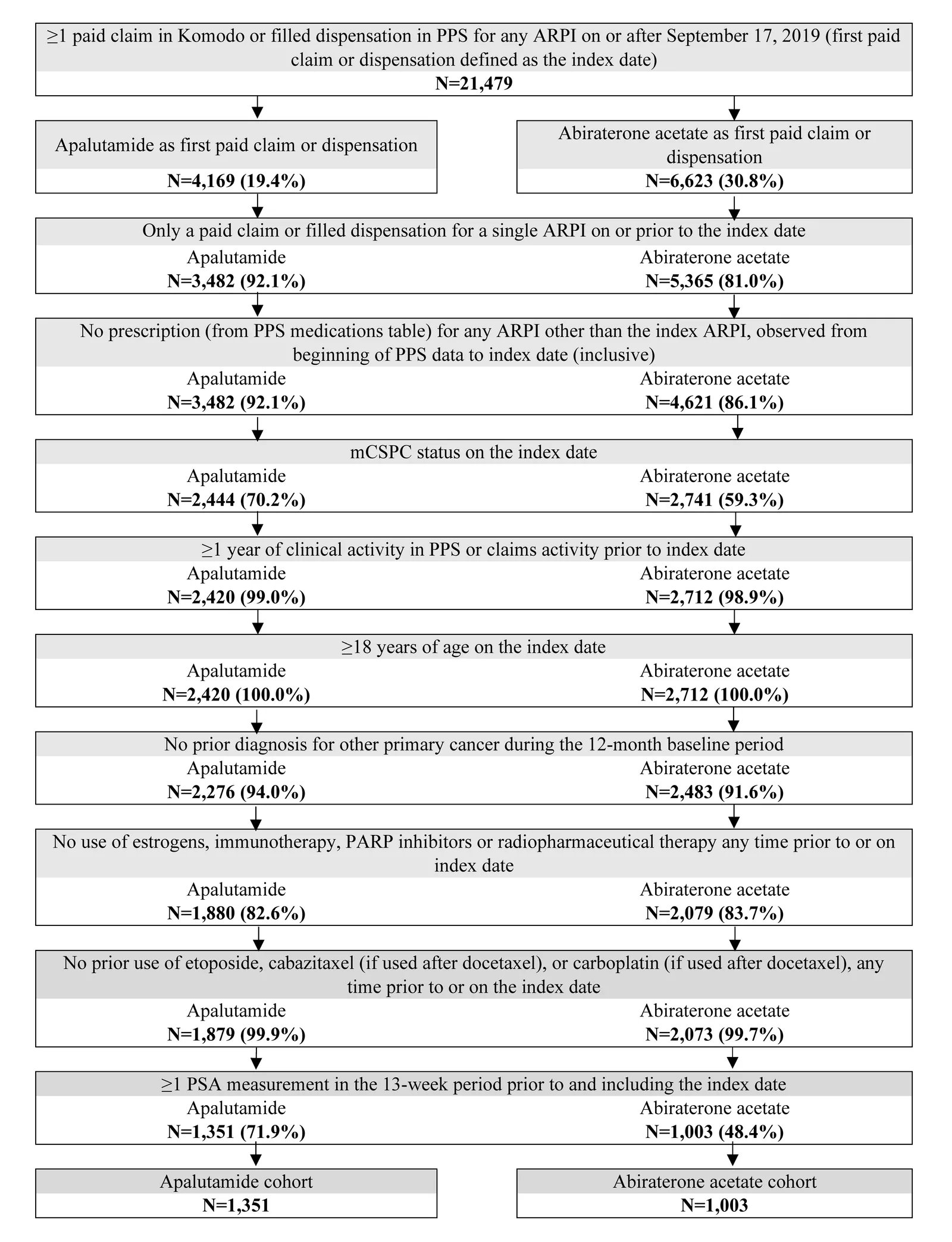
Patient Flowchart Abbreviations: ARPI, androgen receptor pathway inhibitor; mCSPC, metastatic castration-sensitive prostate cancer; PARP, poly ADP-ribose polymerase; PSA, prostate-specific antigen.

### Study Measures

Patient demographics (ie, age, race, geographic region, payer type, and index date) and clinical characteristics (eg, time between PC diagnosis or metastasis and the index date, de novo mCSPC [defined as ≤180 days between first prostate cancer diagnosis and the date of metastasis], prior ADT use, first-generation ARPI use, chemotherapy use, metastasis location [bone, nodal, visceral; not mutually exclusive], the most recent PSA measurement, the most recent testosterone level, and initial Gleason score) were evaluated during the 12-month baseline period.

A PSA90 response was defined by the first follow-up PSA measurement from PPS which showed a ≥90% relative decline from the most recent baseline PSA value observed within the 13-week period prior to or on the index date. The proportion of patients who achieved a PSA90 response by 6 months and 12 months post index was described.

PSA testing patterns were also descriptively reported during the follow-up period, including the proportion of patients with ≥1 PSA test within 3, 6, and 12 months of the index date and the number of follow-up PSA tests per year.

### Statistical Analysis

Inverse probability of treatment weighting (IPTW) based on a propensity score (PS) was used to adjust for differences in the observed baseline characteristics of patients in the apalutamide and abiraterone acetate cohorts.^27^ The PS was estimated using the predicted probability from a single logistic regression model whereby the index treatment received (ie, apalutamide or abiraterone acetate) was the outcome variable and the following pre-index characteristics were defined as predictor variables: age (continuous), race, geographic region, payer type, index year (categorical), time between metastasis and the index date (continuous and categorical), time between the PC diagnosis and the index date (continuous), de novo mCSPC, prior ADT use, first-generation ARPI use, chemotherapy use, metastasis location, the most recent PSA level (continuous), and initial Gleason score (≤6, 7, 8, 9, 10, or unknown).

Each patient was attributed a weight that was defined as 1/(PS) for the apalutamide cohort and 1/(1-PS) for the abiraterone acetate cohort. The normalized inverse probability of treatment weights were truncated at the 95th percentiles to prevent undue influence from patients with outlier weights. No patients were excluded from the study due to the truncation of outlier weights. The balance of baseline characteristics between the two treatment cohorts after IPTW was confirmed by standardized differences <10%.^28^

The proportion of patients in the apalutamide and abiraterone acetate cohorts who achieved a PSA90 response 6 months after the index date was assessed using weighted Kaplan-Meier analyses. A weighted Cox model was used to evaluate the relationship between the index treatment and the likelihood of achieving a PSA90 response at 6 months, which was reported using hazard ratios (HRs) and 95% confidence intervals (CIs). Given the proportional hazards assumption was not met, the model incorporated a time-varying treatment effect via a product interaction term between treatment and log follow-up time. To address the heterogeneity in the frequency and timing of PSA testing between the study cohorts during the follow-up period, a sensitivity analysis was conducted to evaluate PSA90 outcomes among patients with ≥1 follow-up PSA test at 6 months.

## RESULTS

The study sample consisted of 1351 patients with mCSPC who initiated apalutamide and 1003 who initiated abiraterone acetate.

### Baseline Characteristics

After applying IPTW, the baseline characteristics were well balanced between the weighted cohorts, with standardized differences <10% (**Table 1**). The mean (median) age was 72.6 (73.0) in the weighted apalutamide cohort and 72.4 (73.0) in the weighted abiraterone acetate cohort. The proportion of Black patients was 18.7% in the apalutamide cohort and 18.4% in the abiraterone acetate cohort. The most recent mean (median) baseline PSA level was 27.7 (3.6) ng/mL in the apalutamide cohort and 28.1 (3.4) ng/mL in the abiraterone acetate cohort. The median time between metastasis diagnosis and the index treatment initiation was 2.3 months in the apalutamide cohort and 2.3 months in the abiraterone acetate cohort. More than a quarter of the patients initiated ARPI treatment in 2023 in both cohorts (apalutamide: 26.7%; abiraterone acetate: 26.8%)

**Table 1.** Baseline Characteristics

	**Nonweighted Population**			**Weighted Population^a^**		
**Apalutamide (N=1351)**	**Abiraterone Acetate (N = 1003)**	**Standardized Difference, %**	**Apalutamide (N=1351)**	**Abiraterone Acetate (N = 1003)**	**Standardized Difference, %**	
Age, mean ± SD [median]	73.3 ± 8.9 [74.0]	71.2 ± 9.1 [71.0]	23.2	72.6 ± 9.1 [73.0]	72.4 ± 9.0 [73.0]	2.4
Race, n (%)						
White	851 (63.0)	647 (64.5)	3.2	862 (63.8)	641 (63.9)	0.2
Black or African American	285 (21.1)	145 (14.5)	17.4	253 (18.7)	184 (18.4)	0.9
Hispanic or Latino	90 (6.7)	72 (7.2)	2.0	92 (6.8)	67 (6.6)	0.6
Other	44 (3.3)	53 (5.3)	10.0	53 (3.9)	41 (4.1)	1.1
Unknown	81 (6.0)	86 (8.6)	9.9	92 (6.8)	70 (7.0)	0.8
Geographic region, n (%)						
South	767 (56.8)	358 (35.7)	43.3	661 (48.9)	471 (47.0)	3.9
Midwest	303 (22.4)	339 (33.8)	25.5	362 (26.8)	281 (28.0)	2.6
Northeast	149 (11.0)	162 (16.2)	15.0	172 (12.8)	133 (13.2)	1.4
West	132 (9.8)	144 (14.4)	14.1	155 (11.5)	118 (11.8)	1.0
Payer type, n (%)						
Medicare	1071 (79.3)	719 (71.7)	17.7	1038 (76.8)	762 (76.0)	2.0
Commercial	237 (17.5)	246 (24.5)	17.2	269 (19.9)	203 (20.2)	0.7
Medicaid	40 (3.0)	31 (3.1)	0.8	41 (3.0)	29 (2.9)	0.7
Unknown	3 (0.2)	7 (0.7)	0.0	3 (0.2)	9 (0.9)	0.0
Index year, n (%)						
2019-2020	300 (22.2)	217 (21.6)	1.4	299 (22.1)	223 (22.3)	0.3
2021	324 (24.0)	213 (21.2)	6.6	315 (23.3)	232 (23.2)	0.4
2022	367 (27.2)	290 (28.9)	3.9	375 (27.8)	279 (27.8)	0.0
2023	360 (26.6)	283 (28.2)	3.5	361 (26.7)	269 (26.8)	0.1
Time between metastasis and index date, mo, median [Q1, Q3]	2.4 [0.9, 6.4]	2.1 [0.9, 6.2]	11.8	2.3 [0.9, 6.4]	2.3 [0.9, 6.7]	1.1
Time between PC diagnosis and index date, mo, median [Q1, Q3]	16.1 [3.5, 66.2]	7.7 [2.6, 41.1]	22.8	11.5 [3.0, 60.6]	17.1 [3.0, 56.5]	1.1
Metastasis type^b^, n (%)						
Bone	925 (68.5)	593 (59.1)	19.5	881 (65.2)	649 (64.7)	1.1
Nodal	714 (52.8)	609 (60.7)	15.9	754 (55.8)	564 (56.3)	0.9
Visceral	225 (16.7)	201 (20.0)	8.8	238 (17.6)	180 (17.9)	0.8
Metastasis in multiple sites	399 (29.5)	261 (26.0)	7.9	384 (28.4)	283 (28.2)	0.6
Quan-CCI, mean ± SD [median]	8.6 ± 2.8 [9.0]	8.6 ± 2.8 [8.0]	2.0	8.6 ± 2.8 [9.0]	8.6 ± 2.8 [9.0]	0.3
De novo mCSPC^c^, n (%)	717 (53.1)	649 (64.7)	23.8	769 (56.9)	582 (58.0)	2.3
Concurrent use of ADT with index ARPI^d^, n (%)	1,263 (93.5)	894 (89.1)	15.5	1,252 (92.6)	912 (90.9)	6.2
Prior use of first-generation ARPI^e^, n (%)	204 (15.1)	197 (19.6)	12.0	226 (16.8)	174 (17.4)	1.7
Prior use of chemotherapy^f^, n (%)	17 (1.3)	34 (3.4)	14.2	24 (1.8)	23 (2.3)	3.3
Most recent PSA level^g^, ng/mL, mean ± SD [median]	26.2 ± 57.5 [3.5]	30.5 ± 61.5 [3.8]	7.2	27.7 ± 59.6 [3.6]	28.1 ± 59.4 [3.4]	0.7
≤0.2, n (%)	285 (21.1)	202 (20.1)	2.4	282 (20.9)	210 (21.0)	0.3
>0.2 to ≤2, n (%)	274 (20.3)	210 (20.9)	1.6	279 (20.6)	206 (20.5)	0.3
>2 to ≤5, n (%)	188 (13.9)	121 (12.1)	5.5	180 (13.3)	133 (13.3)	0.0
>5 to ≤10, n (%)	159 (11.8)	92 (9.2)	8.5	145 (10.8)	104 (10.4)	1.2
>10, n (%)	445 (32.9)	378 (37.7)	10.0	465 (34.5)	350 (34.9)	0.9
Initial Gleason score^h^, n (%)						
≤6	90 (6.7)	45 (4.5)	9.5	81 (6.0)	57 (5.7)	1.2
7	280 (20.7)	184 (18.3)	6.0	270 (20.0)	200 (20.0)	0.0
8	192 (14.2)	177 (17.6)	9.4	208 (15.4)	158 (15.7)	0.9
9	304 (22.5)	260 (25.9)	8.0	321 (23.8)	243 (24.2)	1.0
10	43 (3.2)	37 (3.7)	2.8	46 (3.4)	34 (3.4)	0.1
Unknown	442 (32.7)	300 (29.9)	6.1	425 (31.4)	311 (31.0)	1.0
Patients with baseline testosterone test, n (%)	897 (66.4)	600 (59.8)	13.7	874 (64.7)	617 (61.5)	6.7
Most recent testosterone level <50 ng/dL, n (%)	627 (69.9)	412 (68.7)	2.7	614 (70.3)	431 (69.9)	0.9

Abbreviations: ADT, androgen deprivation therapy; ARPI, androgen receptor pathway inhibitor; CCI, Charlson Comorbidity Index; mCSPC, metastatic castration-sensitive prostate cancer; PC, prostate cancer; PSA, prostate-specific antigen; Q1, first quartile; Q3, third quartile; SD, standard deviation.

^a^The number of patients reported in this weighted population represents the sum of weights for the corresponding nonweighted patients, rounded to the nearest integer. The proportions displayed were calculated before the rounding and may be slightly different than if they were calculated based on rounded numbers.

^b^Types of metastases were defined at any time prior to (and including) the index date. Types of metastases were not mutually exclusive.

^c^De novo mCSPC was defined as ≤180 days between PC diagnosis and date of metastasis.

^d^Concurrent use was defined as having a record for an ADT agent from 180 days before or after the index date.

^e^Prior use of first-generation ARPI was defined as any prescription for bicalutamide, nilutamide, or flutamide in the 12 months preceding the index date.

^f^Prior chemotherapy use was defined as any administration in the 12 months preceding the index date.

^g^Most recent PSA level was assessed within 13 weeks prior to or on the index date.

^h^Gleason score was evaluated at any time prior to and including the index date.

### PSA Outcomes

The mean (median) on-treatment follow-up period was 11.0 (7.0) months in the apalutamide cohort and 12.1 (9.4) months in the abiraterone acetate cohort. Patients treated with apalutamide attained a descriptively earlier PSA90 response relative to those treated with abiraterone acetate, with a median time to PSA90 response of 3.8 months for apalutamide and 8.7 months for abiraterone acetate.

By 6 months post index, 61.4% of the patients in the weighted apalutamide cohort and 45.9% of those in the weighted abiraterone acetate cohort achieved a PSA90 response (**Figure 3**). Patients who were treated with apalutamide had a 73% higher rate of achieving PSA90 response at 6 months compared with patients who were treated with abiraterone acetate (HR: 1.73; 95% CI, 1.44-2.08; *P* < .001).

**Figure 3.**
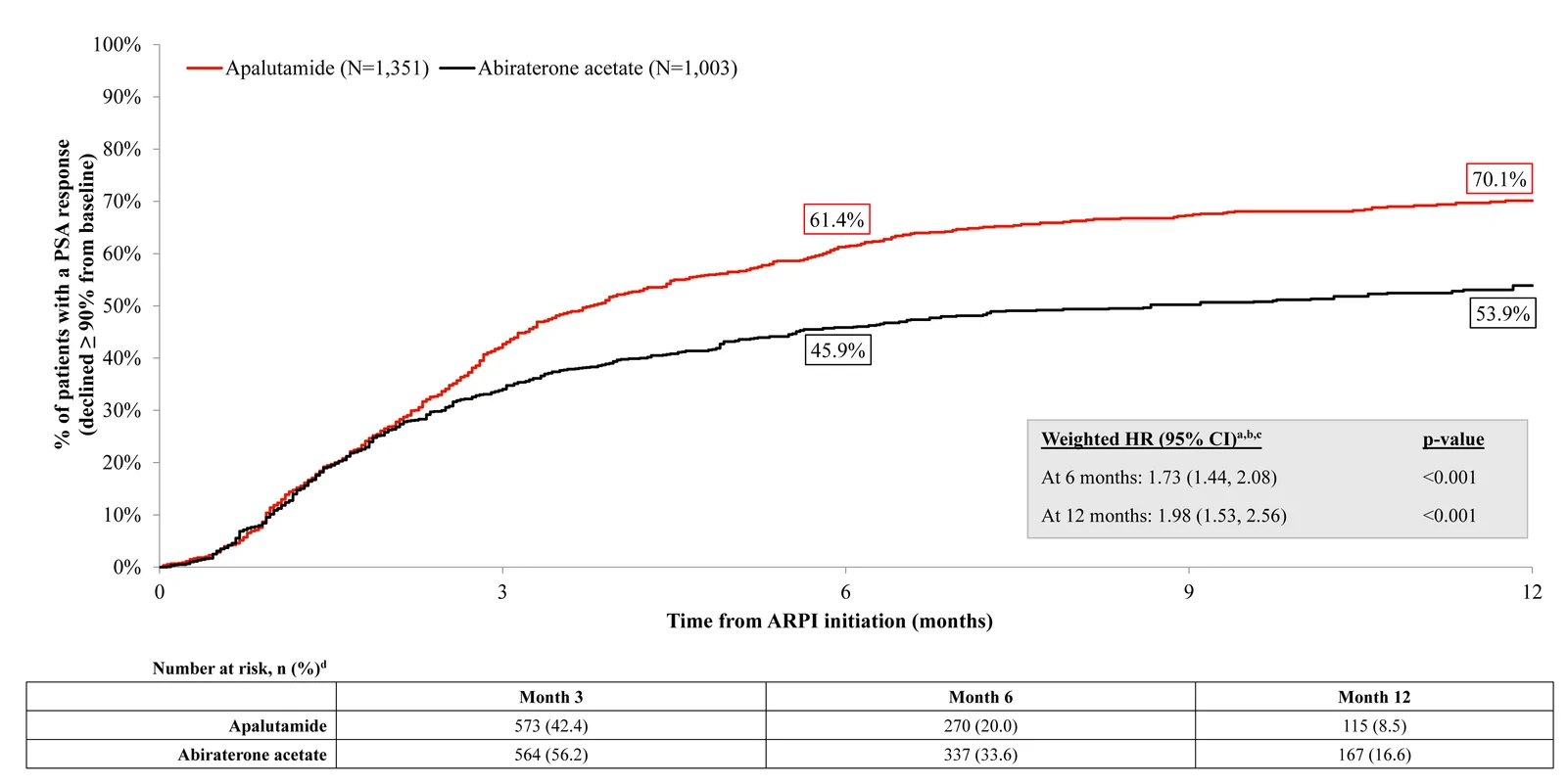
Kaplan-Meier Rates for PSA90 Response Abbreviations: ARPI, androgen receptor pathway inhibitor; CI, confidence interval; HR, hazard ratio; mCSPC, metastatic castration-sensitive prostate cancer; PARP, poly ADP-ribose polymerase; PSA, prostate-specific antigen. ^a^Propensity scores were generated using probability estimates from a logistic regression models using the following predictors: age (continuous), race, geographic region, payer, time between metastasis and index date (continuous and categorical), index year (categorical), time between PC diagnosis and index date (continuous), de novo mCSPC, ADT use overlapping with index date, first-generation ARPI use, chemotherapy use, types of metastases (bone, nodal, visceral, and metastasis in multiple sites), Quan-Charlson comorbidity index (continuous), most recent PSA level (categorical), and earliest Gleason score (categorical). Each patient was attributed an inverse-probability of treatment weight that was defined as follows: 1/(propensity score) for the apalutamide cohort (treated cohort) and 1/(1 − propensity score) for the abiraterone acetate cohort (control cohort). Normalized inverse-probability of treatment weights were truncated at the 95th percentiles. ^b^A hazard ratio >1 indicates that the apalutamide cohort had a higher rate of PSA90 compared with the abiraterone acetate cohort. ^c^Given the proportional hazards assumption was not met, the model incorporated a time-varying treatment effect via a product interaction term between treatment and log follow-up time. ^d^The number of patients reported in this weighted population represents the sum of weights for the corresponding nonweighted patients, rounded to the nearest integer. The proportions displayed were calculated before the rounding and may be slightly different than if they were calculated based on rounded numbers.

Results were consistent by 12 months post index, with 70.1% of patients in the weighted apalutamide cohort and 53.9% of those in the weighted abiraterone acetate cohort achieving a PSA90 response. Patients treated with apalutamide had a 98% higher rate of achieving PSA90 response at 12 months compared with patients treated with abiraterone acetate (HR, 1.98; 95% CI, 1.53-2.56; *P* < .001). Results from the sensitivity analysis among patients with ≥1 follow-up PSA test at 6 months (1073 patients in the apalutamide cohort and 790 patients in the abiraterone acetate cohort) were consistent with the main analysis (time-varying HR, 1.62; *P* < .001).

### PSA-Related Testing

The frequency of PSA testing during the follow-up period was similar between patients in the weighted apalutamide cohort (80.5%) and the weighted abiraterone acetate cohort (81.2%), with most patients having ≥1 follow-up PSA measurement by 6 months post index (78.8% and 78.9%, respectively; **Table 2**) and by 12 months post-index (80.4% and 80.9%, respectively). The mean (median) number of follow-up PSA tests per year was 4.0 (3.7) in the apalutamide cohort and 4.8 (4.2) in the abiraterone acetate cohort.

**Table 2.** Follow-up PSA Testing

	**Nonweighted Population**		**Weighted Population^a^**	
**Apalutamide (N = 1351)**	**Abiraterone Acetate (N = 1003)**	**Apalutamide (N = 1351)**	**Abiraterone Acetate (N = 1003)**	
Patients with ≥1 PSA test, n (%)	1096 (81.1)	812 (81.0)	1088 (80.5)	815 (81.2)
Within first 3 mo of follow-up	950 (70.3)	712 (70.9)	943 (69.8)	706 (70.4)
Within first 6 mo of follow-up	1073 (79.4)	790 (78.8)	1065 (78.6)	792 (78.9)
Within first 12 mo of follow-up	1094 (81.0)	809 (80.7)	1086 (80.4)	811 (80.9)
No. of follow-up PSA tests per year, mean ± SD [median]	4.1 ± 3.5 [3.7]	4.8 ± 4.2 [4.2]	4.0 ± 3.5 [3.7]	4.8 ± 4.2 [4.2]
Patients with PSA test on average every 3 mo, n (%)	581 (43.0)	531 (52.9)	573 (42.4)	528 (52.6)
Patients with PSA test on average every 6 mo, n (%)	1023 (75.7)	732 (73.0)	1016 (75.2)	739 (73.7)

Abbreviations: PSA, prostate-specific antigen; SD, standard deviation.

^a^The number of patients reported in this weighted population represents the sum of weights for the corresponding nonweighted patients, rounded to the nearest integer. The proportions displayed were calculated before the rounding and may be slightly different than if they were calculated based on rounded numbers.

## DISCUSSION

Using clinical data linked with administrative claims, this real-world study evaluated PSA outcomes among patients with mCSPC in the US who were treated with apalutamide or abiraterone acetate between 2019 and 2023. Findings show that patients treated with apalutamide attained a PSA90 response nearly 5 months earlier relative to those treated with abiraterone acetate. At 6 months after treatment initiation, patients who received apalutamide were 73% more likely to achieve a PSA90 response compared with patients who received abiraterone acetate.

By incorporating more than a year of additional data and nearly 800 additional patients, this study confirms the findings of the prior analysis by Brown et al, which showed that patients with mCSPC who initiated apalutamide were significantly more likely to achieve a PSA90 response after 6 months of treatment compared with those who initiated abiraterone acetate (63.9% and 41.7%, respectively, achieved PSA90 by 6 months in Brown et al).^26^ An important strength of both studies is the linking of the EMR from PPS Analytics, which provided clinical and in-office dispensing data from community-based urology practices, with administrative claims from the KRD, which included information on filled prescriptions to verify the receipt of the index treatment. In the absence of specific diagnosis codes for mCSPC in the medical claims, the use of EMR data permitted the selection of patients with mCRPC for exclusion from the study population by incorporating clinical information available for all patients, supplemented by several indicators as a proxy for castration resistance (eg, rise in PSA while medically or surgically castrate), which enhanced the patient selection process. Given the expanding landscape of available ARPI treatments for patients with mCSPC and the lack of head-to-head RCT comparing them, these large, real-world comparative studies provide the information necessary to guide treatment decision-making in clinical practice.

Although there are no head-to-head RCTs evaluating PSA response in patients treated with apalutamide vs abiraterone acetate, the proportion of patients who achieved a PSA90 response after 6 months of apalutamide treatment in the current study (61.4%) was consistent with the proportion observed in the TITAN study (63%)^29^ (this information has not been reported for patients who received abiraterone acetate in the LATITUDE trial). Some real-world studies, mainly with limited sample sizes, have also provided comparative data on the effectiveness of apalutamide vs abiraterone acetate in reducing PSA levels, showing a significant benefit with apalutamide.^24,30^ In the recent single-center study by Pons-Fuster et al, patients with mCSPC treated with apalutamide (n = 65) had a significantly longer duration of PSA progression-free survival (defined as the time from treatment initiation to the first increase in PSA levels exceeding 25% and 2 ng/mL above nadir) compared with patients treated with abiraterone acetate (n = 39; *P* = .015) with 19% and 41% of patients, respectively, experiencing PSA progression during the study.^24^ Moreover, there was a significant difference between cohorts in the proportion of patients who achieved a PSA90 response as early as the first month of treatment, which was 63.5% in the apalutamide cohort and 28.2% in the abiraterone acetate cohort (*P* < .001).^24^ Another retrospective analysis by Maughan et al used EMR from US oncology clinics to show that after 6 months of treatment, 38.7% of patients with mCSPC in the apalutamide cohort (n = 315) experienced PSA progression relative to 45.0% of the abiraterone acetate cohort (n = 1760), with patients in the apalutamide cohort being 30% more likely to achieve a PSA90 response at any time compared with abiraterone acetate (HR, 1.3; 95% CI, 1.1-2.6; *P* < .001).^30^ The results of this study confirm these findings in a robust study sample and support the real-world benefit of apalutamide in inducing an earlier and deeper PSA response among patients with mCSPC compared with abiraterone acetate.

Importantly, the high rate and rapid onset of the PSA90 response associated with apalutamide over abiraterone acetate among patients with mCSPC can have a meaningful prognostic impact. Indeed, several studies have confirmed the association of an early and deep PSA response with survival-related endpoints among patients with mCSPC.^16–23^ For example, in the multicenter real-world ARON-3 study, patients with mCSPC who received apalutamide and achieved a PSA90 response (57% of whom achieved this response within 6 months) had a significantly lower 24-month mortality rate than patients who did not achieve a PSA90 response (*P* < .001).^22^ Similarly, a recent analysis of linked EMR data from PPS with administrative claims data from the KRD showed that an early and deep PSA response was an indicator of long-term survival and treatment success among patients with mCSPC initiating ARPIs in the real world.^23^ Specifically, patients with mCSPC treated with ARPIs who achieved a PSA90 response by 6 months had significantly better overall survival by 36 months after treatment initiation relative to those who did not (HR, 0.40; 95% CI, 0.31-0.50; *P* < .001).^23^ Using the same linked data sources, a comparative analysis of real-world survival outcomes among patients with mCSPC showed that those treated with apalutamide had a 26% lower mortality rate by 24 months post treatment initiation compared with those who received abiraterone acetate (HR, 0.74; 95% CI, 0.59-0.93; *P* = .010).^15^ Given that there are no head-to-head trials comparing apalutamide and abiraterone acetate among patients with mCSPC, the findings from the current study can be considered in addition to the existing literature to help inform treatment decisions in patients initiating APRIs. The implications of these findings warrant important consideration in clinical practice and underscore the value of achieving an early PSA response for patients with mCSPC treated with ARPIs to improve their long-term survival outcomes. Furthermore, the real-world benefit of apalutamide for the treatment of mCSPC has been demonstrated in prior research using other clinical outcomes not evaluated in the current study, including PSA50 response (ie, ≥50% decrease in PSA level from the most recent pre-treatment PSA level) and time to castration resistance, in addition to being associated with high levels of real-world adherence and treatment persistence.^31,32^

### Limitations

The interpretation of the study results is subject to certain limitations. Although the baseline characteristics were well balanced between the study cohorts after applying IPTW, some differences were not accounted for. For example, unlike apalutamide, abiraterone acetate is only indicated for the treatment of patients with high-risk mCSPC and disease volume was not available in the data and could not be balanced using IPTW. However, visceral metastasis and metastasis in multiple sites, which are related to high-volume disease, were balanced between cohorts. Nevertheless, future studies incorporating disease volume and excluding patients with unknown testosterone levels would be needed to corroborate the study findings. Additionally, the heterogeneity in the frequency and timing of PSA testing between the study cohorts during the follow-up period may have resulted in a surveillance bias and affected the study results. To address this limitation, a sensitivity analysis was conducted to evaluate PSA90 outcomes among patients with ≥1 follow-up PSA test at 6 months and found similar results, supporting the robustness of the study findings. Inherent to claims-based studies, potential selection or information biases due to miscoding or misclassification in the EMR data or administrative claims may have impacted the analyses. While, only observed confounders could be included to adjust between cohorts, the covariates that were included in the IPTW model were comprehensive and covered known predictors of PSA90 response in patients with mCSPC treated with ARPIs. Finally, although a link between a deep and rapid PSA response and improved long-term prognosis has been established in patients with mCSPC,^16–23^ this study did not directly evaluate the association between PSA90 and overall survival in this population.

## CONCLUSIONS

This retrospective study provides robust evidence comparing rapid PSA90 response among patients with mCSPC treated with apalutamide vs abiraterone acetate in real-world clinical practices in the US. The results show that apalutamide was associated with a faster and significantly higher rate of 6-month PSA90 response compared with abiraterone acetate. Considering the previously established relationship between the attainment of a rapid and deep PSA response with long-term survival outcomes in this patient population,^16–23^ the findings of this study may represent an important factor in guiding the selection of frontline ARPI treatment for patients with mCSPC.

### Disclosures

This study was supported by Johnson & Johnson. B.L. is an employee of Chesapeake Urology Associates and has received consulting fees from Johnson & Johnson. M.B. is an employee of the Winship Cancer Institute of Emory University and has received consulting fees from Johnson & Johnson. S.B., C.P. are employees and stockholders of Johnson & Johnson. F.K., C.R., G.W., and D.P. are employees of Analysis Group, Inc., a consulting company that has provided paid consulting services to Johnson & Johnson. N.S. is an employee of START Carolinas/Carolina Urologic Research Center and has received consulting fees from Johnson & Johnson.
